# Anaesthesia generates neuronal insulin resistance by inducing hypothermia

**DOI:** 10.1186/1471-2202-9-100

**Published:** 2008-10-09

**Authors:** Christian Holscher, Lidy van Aalten, Calum Sutherland

**Affiliations:** 1 Biomedical Research Institute, University of Dundee, Ninewells Hospital, Dundee, Scotland, DD1 9SY, UK; 2 School of Biomedical Sciences, University of Ulster, Coleraine, BT52 1SA, Northern Ireland, UK

## Abstract

**Background:**

Anaesthesia is commonly employed prior to surgical investigations and to permit icv injections in rodents. Indeed it is standard practise in many studies examining the subsequent actions of hormones and growth factors on the brain. Recent evidence that the basal activity of specific intracellular signalling proteins can be affected by anaesthesia prompted us to examine the effect of anaesthesia not only on the basal activity but also the insulin sensitivity of the major insulin signalling pathways.

**Results:**

We find that urethane- and ketamine-induced anaesthesia results in rapid activation of the phosphatidylinositol (PI) 3-kinase-protein kinase B (PKB) signalling pathway in the brain, increases tau phosphorylation while at the same time reducing basal activity of the Ras-ERK pathway. Subsequent injection of insulin does not alter the activity of either the PI 3-kinase or ERK signalling pathways, indicating a degree of neuronal molecular insulin resistance. However, if body temperature is maintained during anaesthesia then there is no alteration in the basal activity of these signalling molecules. Subsequent response of both pathways to insulin injection is restored.

**Conclusion:**

The data is consistent with a hypothermia related alteration in neuronal signalling following anaesthesia, and emphasises the importance of maintaining the body temperature of rodents when monitoring insulin (or growth factor/neurotrophic agent) action in the brain of anesthetised rodents.

## Background

Insulin is produced by pancreatic β-cells, in response to rising plasma glucose levels, and initiates multiple metabolic changes to restore glucose homeostasis. A specific membrane glycoprotein acts as a high affinity sensor for insulin (insulin receptor (IR)) in many tissues (primarily liver, fat, muscle). The IR is expressed in many regions of the brain, including the hypothalamus, cortex, and hippocampus. Neuron specific deletion of the IR makes the animal more sensitive to diet induced obesity [1], implicating neuronal IR in the satiety response. Consistent with this, administration of insulin to the arcuate nucleus in the hypothalamus has significant effects on feeding and body weight [2-4].

Epidemiological evidence suggests that whole body insulin resistance, related to obesity, increases the risk of Alzheimer's disease, as well as vascular dementia [5-7]. In addition, there are molecular links between the development of Type 2 diabetes (an insulin resistant state) and Alzheimers's disease [8]. For example, GSK3β activity is increased in the muscle of Type 2 diabetics [9] and the brain of Alzheimer's patients [10]. This enzyme is inhibited by insulin treatment of cells [11], and is known to be a major tau kinase, phosphorylating residues that become hyperphosphorylated in Alzheimer's disease [12]. Therefore, insulin resistance in the brain may contribute to a major component of Alzheimer's disease pathology.

Binding of insulin to the IR activates the intrinsic tyrosine kinase domain of the IR [13,14]. Insulin receptor substrate (IRS) proteins are recruited to the activated IR, become phosphorylated on tyrosine residues [13], thereby recruiting PI 3-kinase, which converts phosphoinositol 4,5 bisphosphate (PIP2) to phosphoinositol 3,4,5 trisphosphate (PIP3) [15]. This second messenger then brings pleckstrin homology (PH) domain containing proteins to the membrane, activating protein kinase cascades. The best characterised of these is the phosphoinositide dependent protein kinase (PDK1) pathway. PDK1 regulates many protein kinases, including protein kinase B (PKB, also known as Akt), PKC, p90 RSK, p70S6K and SGK [16]. These protein kinases phosphorylate and regulate a wide variety of proteins involved in growth and metabolism. For example, PKB phosphorylates and inactivates GSK3 [11], and FOXO transcription factors [17]. These pathways are important for the proper regulation of hepatic gene transcription by insulin [18,19].

The second major pathway downstream of IRSs is the Ras-ERK pathway. Grb2/mSOS is a protein complex that interacts with phosphorylated IRSs (at distinct residues to those that recruit PI 3-kinase). Once bound, mSOS exchanges GDP for GTP on the small G-protein Ras, thereby activating Ras [20]. This promotes activation of c-Raf, which phosphorylates and activates MAP/ERK kinase (MEK), which in turn phosphorylates and activates ERK1/2 [20-22]. ERK1/2 has multiple substrates, most of which are related to cell growth, hence this pathway is generally considered to be important in insulin regulation of growth.

Most studies examining insulin action in the brain utilise primary neurons, slice culture or transgenic animals. However, direct application of insulin to the brain, by intracerebroventricular (icv) injection, can be used to study acute and chronic effects of insulin *in vivo*. Recent evidence suggests that some of the signalling molecules described above are affected by anaesthesia [23,24], which is commonly used prior to icv injection. In this report we examined the two major insulin signalling pathways in the brain of anaesthetised rodents, and find that anaesthesia induced hypothermia generates insulin resistance in the brain.

## Methods

### Anaesthesia and ICV injections

Male C57/Bl6 mice 3 months of age were obtained from Harlan, UK. All studies were performed under the regulations permitted by UK home office licence no. PPL2603b. Animals were fasted overnight and anesthetised by ip injection of either urethane (750 mg/kg dose in 0.2 ml) or ketamine/Xylazine (80–100 mg/kg + 10 mg/kg).

For some studies (as indicated in figure legends) anesthetised animals were kept at 37°C by use of a temperature controlled heating pad (Harvard apparatus, rectal probe). For icv injection of insulin (3 mU in 2 μl), animals were injected with ketamine/Xylazine, put into a stereotaxic frame (TSE systems, Germany), the scalp was removed, and a 0.7 mm hole was drilled to permit injection into the lateral ventricle using a 5 μl Hamilton syringe (coordinates from bregma: AP = 0.2, ML = 1.2, D = 2.5). Animals were injected immediately with sterile saline solution (control) or insulin. The injection was given slowly over 2 minutes. Body temperature was approximately 34°C by the time of the icv injection.

After 30 min, animals were decapitated, the brains removed and dissected into different brain sections. Brain sections were then snap frozen in dry ice and stored at -40 until analysis. Non-anesthetised controls were injected ip with saline and were decapitated after 30 min without any surgery, and the brains removed and snap frozen.

### Antibodies

Antibodies to phospho-PKB (Thr308), phospho-PKB (Ser473), phospho-ERK (Thr202/Tyr204), were from Cell Signaling Technology (Beverly, MA, U.S.A.), antibodies to total PKB, ERK1/2 and GSK3α/β were from Upstate Biotechnologies (Lake Placid, NY, U.S.A.). Anti-β-Actin was purchased from Sigma-Aldrich, Inc. (St Louis, MO, U.S.A.). The phospho-tau (AT8) and tau-5 (total tau) antibodies were purchased from Innogenetics (Gent, Belgium) and Chemicon, respectively.

### Tissue Homogenisation and Immunoblot

Frozen tissues were homogenised using a 1 ml glass Dounce homogenizer in lysis buffer containing 1% (v/v) Triton X-100, 50 mM Tris-HCl, pH 7.5, 0.27 M sucrose, 1 mM sodium orthovanadate, 0.1% (v/v) β-mercaptoethanol and Complete protease inhibitor tablets (Roche, Lewes, UK) (4°C). Following centrifugation, supernatants were collected and protein concentrations determined [25]. Lysates (typically 2.5–30 μg) were subjected to SDS-PAGE on 4–12% NuPAGE polyacrylamide gels, then transferred to nitrocellulose membrane. Membranes were incubated with primary antibodies (diluted 1/1000 in 1% (w/v) skimmed milk or 5% (w/v) BSA in TTBS overnight at 4°C), appropriate HRP-linked secondary antibodies, then visualised using the ECL reagent (GE Healthcare) and exposed to autorad film (GE Healthcare).

### Analysis of data

Densitometry was performed using Aida computer software (Raytest, Straubenhardt, Germany). Phosphorylation of ERK, Akt and tau were calculated as the ratio of the phosphospecific versus total protein antibody signal, while phosphorylation of GSK3 was a ratio of the P-GSK3 to β-actin (loading control). Mean values were calculated for each sample and the control value was set at 1. Data is presented as mean ± standard deviation, and statistical significance (p-value) between two conditions was obtained using an unpaired students t-test.

## Results and discussion

### Anaesthesia induces changes in the major insulin regulated protein kinase cascades resulting in loss of subsequent response to insulin

**Figure 1:**
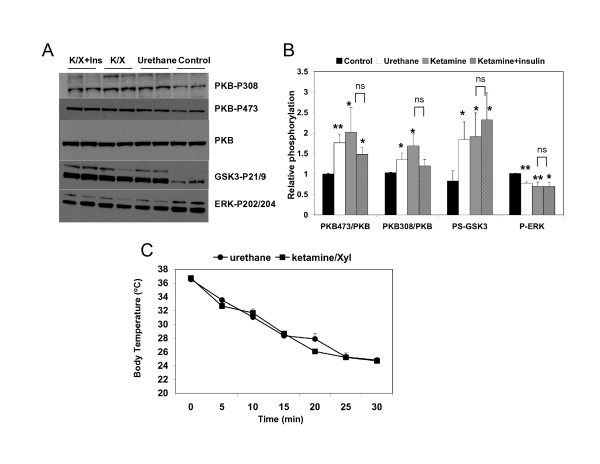
**Anaesthesia induces neuronal insulin resistance**. Mice were injected i.p. with either urethane or ketamine/Xylazine (K/X). Half of the K/X group were given insulin by icv injection, the other half received saline, 30 mins prior to sacrifice and rapid removal and dissection of the brain. Control animals were sacrificed without anaesthesia or icv injection. Equal amounts of cortical protein were subjected to western blot using the specific antibodies shown (A-representative blots), and results from 4 animals in each treatment group were quantified by densitometry (B). The mean ± SD, relative to the control animals, is shown. Significant differences between control and each treatment group are given, *p < 0.05, **p < 0.005, ns, not significant. Body temperature of all animals during anaesthesia was monitored and is presented as mean ± SD (C).

Eight mice were anesthetised with Ketamine/Xylazine, and four were sacrificed without anaesthesia (Fig. 1). Half of the Ketamine/Xylazine group were injected icv with 3 mU of insulin and the rest with an equivalent volume of saline. The phosphorylation of protein kinase B (PKB) at both Ser-308 and Thr-473 is used as a surrogate for activation [26]. Meanwhile the phosphorylation of the PKB target, GSK3β (at Ser-9), is a measure of intracellular PKB activation [11]. ERK1/2 phosphorylation at Thr-202/Tyr-204 provides a measure of the activity state of this key enzyme [21]. Surprisingly, we found no significant effect of insulin on these pathways in the cortex (Fig. 1) or hippocampus (data not shown). However, the level of phosphorylation of PKB and GSK3 was significantly higher in the anesthetised animals, compared to animals that had been sacrificed without anaesthesia (Fig. 1). The same abnormally high signalling was observed with either ketamine or urethane administration (Fig. 1). This suggests increased basal PKB activity in the anaesthetised animals. In contrast, the phosphorylation of ERK1/2 decreased following anaesthesia (Fig. 1), but as with the PKB pathway, there was no subsequent regulation of ERK by insulin. This suggested that the anaesthesia itself was altering the signalling pathways in a manner that prevented regulation by insulin. Reduced ERK and increased GSK3 phosphorylation have been reported previously in rodents exposed to various anaesthetics [23,24]. However, this is the first demonstration of abnormal phosphorylation of PKB and importantly, of subsequent loss of response of all of these pathways to insulin. The molecular mechanism(s) leading from anaesthesia to induction of PKB activity and repression of ERK activity is not entirely clear. Reduced activity of the protein phosphatase, PP2A, has been found in rodents anesthetised by pentobarbital, and this could be prevented by maintaining body temperature [24]. The increased PKB and GSK3 phosphorylation may be explained by reduced PP2A activity but it is more difficult to understand the lower ERK phosphorylation. Most growth factors (and insulin) will induce both of these pathways, however, dysregulation of the Ras-ERK pathway has been observed in polycystic ovarian syndrome (PCOS) ([27] and CS unpublished data), which is characterised by hyperandrogenism and insulin resistance. The molecular pathology of the defective ERK signalling in PCOS remains to be determined, and indeed it is still unclear whether the insulin resistance precedes or is subsequent to the abnormal regulation of ERK in PCOS.

The previous reports of abnormal signalling processes in anaesthesia had indicated that the induction of hypothermia contributed to the defective signalling. Indeed, simply lowering body temperature could alter ERK and GSK3 signalling [24]. The body temperature of the animals injected with either agent was substantially and reproducibly lowered within the timeframe of the experiment (Fig. 1C). Although it is quite likely that many other physiological processes (including hypoxia) will be induced by anaesthesia, we investigated whether maintenance of body temperature of the anesthetised animals prevented the defective response of PKB, GSK3 or ERK1/2 phosphorylation to insulin.

### Maintenance of body temperature during anaesthesia is required to permit intracellular responses to insulin in the brain

**Figure 2:**
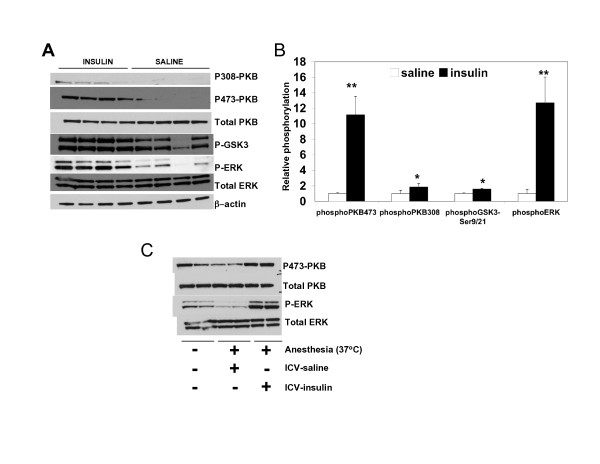
**Insulin sensitivity is maintained during anaesthesia if hypothermia is prevented**. Mice were injected i.p. with ketamine/Xylazine (K/X), and body temperature maintained at 37°C. Half of the group (n = 4) were given insulin by icv injection, the other half (n = 4) received saline 30 mins prior to sacrifice and rapid removal and dissection of the brain. Equal amounts of cortical protein were subjected to western blot using the antibodies shown (A), and results from each treatment group quantified by densitometry (B). The mean ± SD, relative to the control animals, is shown. Significant differences between control and each treatment group are given, *p < 0.05, **p < 0.005. Phosphorylation of ERK and PKB was also compared between animals sacrificed without anaesthesia and those with anaesthesia but whose body temperature was maintained at 37°C (C).

There was a significant insulin induction of both of the PKB-GSK3 and Ras-ERK pathways in the cortex and hippocampus of the anesthetised animals when body temperature was maintained at 37°C (Fig. 2). Significant increases in phosphorylation of Thr308 and Ser473 of PKB, Ser21/9 of GSK3 and Thr202/Tyr204 of ERK were detected following insulin administration (Fig. 2B), while the changes in basal phosphorylation of ERK and PKB were not observed in anaesthetised animals when body temperature was maintained (Fig. 2C). Therefore, the maintenance of body temperature during anaesthesia is absolutely required for insulin activation of these signalling pathways.

This is of particular importance since prolonged insulin resistance in the brain is likely to underlie the increased risk of dementia in people with Type 2 diabetes (for review see [8]). The insulin resistance generated by anaesthesia-associated hypothermia may provide a model system to study the neurodegenerative effects of insulin resistance and/or the neuroprotective effects of insulin, in vivo. Post-operative cognitive defects have been widely reported, particularly in the elderly, and these may be related to general anaesthesia [28,29]. Meanwhile, obesity induced insulin resistance leads to cognitive impairment in rodents [30,31]. Therefore insulin resistance in the brain during anaesthesia may play an important role in postoperative cognitive decline, although it is unlikely that body temperature falls below 30°C in clinical practice, therefore further study is required to establish the exact point where hypothermia is problematic.

The data presented shows the importance of maintaining body temperature in rodents undergoing anaesthesia prior to studies investigating hormonal or growth factor regulation of cognition. The signalling molecules affected by hypothermia are not only key to the action of insulin in the brain, but also the action of neurotrophic agents and other circulating hormones (e.g. Leptin) [4].

### The phosphorylation of tau at Ser202/Thr205 is induced by anaesthesia but remains sensitive to insulin

**Figure 3:**
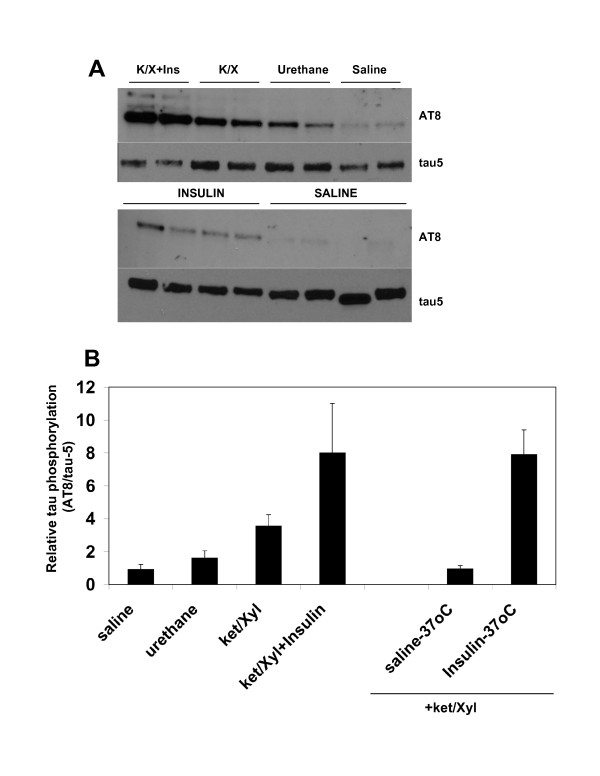
**Tau phosphorylation is increased by anaesthesia but remains partially sensitive to insulin even during hypothermia**. Samples described in Fig. 2 and Fig. 3 were analysed for phosphorylation of tau (A-representative blots) at Ser202 and Thr205 (AT8 antibody), and for total tau (tau-5 antibody). The results from each treatment group were quantified by densitometry (B). The mean ± SD, relative to the control animals, is shown. Significant differences between control and each treatment group are given, *p < 0.05, **p < 0.005.

Hyperphosphorylation of tau, on numerous residues, associates with the development of Alzheimer's disease and other forms of dementia (tauopathies). Previous work demonstrated that tau phosphorylation increases during anaesthesia [24]. We found that tau phosphorylation (at Ser202/Thr205) was increased by urethane or ketamine (Fig. 3). However, in contrast to the complete loss of insulin activation of the PKB and ERK pathways, there was an additive induction of tau phosphorylation following insulin in the anaesthetised animals (Fig. 3). This suggests that these residues do not become completely phosphorylated during anaesthesia, and that insulin regulation of this site is not going through the ERK or PKB pathways. The extent of tau phosphorylation in response to insulin was greater when body temperature was maintained, presumably due to the lower basal phosphorylation (Fig. 3B). Insulin resistance has been implicated as an 'accelerator' of Alzheimer's pathology. The anaesthetised animal may provide a model to establish which phosphorylation events on tau are regulated by the ERK and PKB pathways in response to insulin, and whether anaesthesia worsens the pathology in models of Alzheimer's disease.

## Conclusion

Anaesthesia induces abnormal signalling in the brain, but this can be prevented by maintaining body temperature. The abnormal signalling produces a form of molecular insulin resistance, although this is not equally severe in all insulin signalling pathways. Anaesthesia/hypothermia has potential as an in vivo model for the study of the effect of insulin resistance in the brain. Meanwhile great care must be taken to maintain body temperature when using anesthetised rodents to study neuronal signalling.

## Authors' contributions

CH performed all of the animal procedures, including anaesthesia, icv injections and tissue isolation. LVA performed all of the molecular analysis. The study was conceived by CH and CS, and CS wrote the manuscript and supervised the molecular work.

## References

[B1] Bruning JC, Gautam D, Burks DJ, Gillette J, Schubert M, Orban PC, Klein R, Krone W, Muller-Wieland D, Kahn CR (2000). Role of brain insulin receptor in control of body weight and reproduction. Science.

[B2] Niswender KD, Schwartz MW (2003). Insulin and leptin revisited: adiposity signals with overlapping physiological and intracellular signaling capabilities. Front Neuroendocrinol.

[B3] Niswender KD, Baskin DG, Schwartz MW (2004). Insulin and its evolving partnership with leptin in the hypothalamic control of energy homeostasis. Trends Endocrinol Metab.

[B4] Sutherland C, Ashford ML, D Withers JHa (2008). Leptin and insulin signalling networks. Neurobiology of Obesity.

[B5] Allen KV, Frier BM, Strachan MWJ (2004). The relationship between T2DM and cognitive dysfunction: longitudinal studies and their methodological limitations. Eur J Pharmacol.

[B6] Arvanitakis Z, Wilson RS, Bienias JL, Evans DA, Bennett DA (2004). Diabetes mellitus and risk of Alzheimer disease and decline in cognitive function. Archives of Neurology.

[B7] Yaffe K, Blackwell T, Kanaya AM, Davidowitz N, Barrett-Connor E, Kruegeer K (2004). Diabetes, impaired fasting glucose, and development of cognitive impairment in older women. Neurology.

[B8] Cole A, Astell A, Green C, Sutherland C (2007). Molecular connections between dementia and diabetes. Neuroscience and Biobehavioral Reviews.

[B9] Nikoulina SE, Ciaraldi TP, Mudaliar S, Carter L, Johnson K, Henry RR (2002). Inhibition of glycogen synthase kinase 3 improves insulin action and glucose metabolism in human skeletal muscle. Diabetes.

[B10] Leroy K, Yilmaz Z, Brion JP (2007). Increased level of active GSK-3beta in Alzheimer's disease and accumulation in argyrophilic grains and in neurones at different stages of neurofibrillary degeneration. Neuropathol Appl Neurobiol.

[B11] Cross DAE, Alessi DR, Cohen P, Andjelkovich M, Hemmings BA (1995). Inhibition of GSK3 by insulin mediated by protein kinase B. Nature.

[B12] Ishiguro K, Shiratsuchi A, Sato S, Omori A, Arioka M, Kobayashi S, Uchida T, Imahori K (1993). Glycogen synthase kinase 3 beta is identical to tau protein kinase I generating several epitopes of paired helical filaments. FEBS Lett.

[B13] White MF (1998). The IRS-signalling system: a network of docking proteins that mediate insulin action. Mol Cell Biochem.

[B14] Withers DJ, White M (2000). Perspective: The Insulin Signaling System – A Common Link in the Pathogenesis of Type 2 Diabetes. Endocrinology.

[B15] Vanhaesebroeck B, Leevers SJ, Ahmadi K, Timms J, Katso R, Driscoll PC, Woscholski R, Parker PJ, Waterfield MD (2001). Synthesis and function of 3-phosphorylated inositol lipids. Annu Rev Biochem.

[B16] Mora A, Komander D, van Aalten DM, Alessi DR (2004). PDK1, the master regulator of AGC kinase signal transduction. Semin Cell Dev Biol.

[B17] Rena G, Woods YL, Prescott AR, Peggie M, Unterman TG, Williams MR, Cohen P (2002). Two novel phosphorylation sites on FKHR that are critical for its nuclear exclusion. EMBO J.

[B18] Lochhead PA, Coghlan MP, Rice SQ, Sutherland C (2001). Inhibition of GSK3 selectively reduces G6Pase and PEPCK gene expression. Diabetes.

[B19] Onuma H, Kooi BT Vander, Boustead JN, Oeser JK, O'Brien RM (2006). Correlation between FOXO1 (FKHR) and FOXO3a (FKHRL1) binding and the inhibition of basal glucose-6-phosphatase catalytic subunit gene transcription by insulin. Mol Endocrinol.

[B20] McKay MM, Morrison DK (2007). Integrating signals from RTKs to ERK/MAPK. Oncogene.

[B21] Payne DM, Rossomando AJ, Martino P, Erickson AK, Her J, Shabanowitz J, Hunt DF, Weber M, Sturgill TW (1991). Identification of the regulatory phosphorylation sites in pp42/mitogen-activated protein kinase (MAP kinase). EMBO J.

[B22] Kyriakis JM, App H, Zhang X, Banerjee P, Brautigan DL, Rapp UR, Avruch J (1992). Raf-1 activates MAP kinase-kinase. Nature.

[B23] Li X, Friedman AB, Roh MS, Jope RS (2005). Anesthesia and post-mortem interval profoundly influence the regulatory serine phosphorylation of glycogen synthase kinase-3 in mouse brain. J Neurochem.

[B24] Planel E, Richter KE, Nolan CE, Finley JE, Liu L, Wen Y, Krishnamurthy P, Herman M, Wang L, Schachter JB (2007). Anesthesia leads to tau hyperphosphorylation through inhibition of phosphatase activity by hypothermia. J Neurosci.

[B25] Bradford MM (1976). A rapid and sensistive method for the quantitation of microgram quantities of protein utilising the principle of protein-dye binding. Anal Biochem.

[B26] Hanada M, Feng J, Hemmings BA (2004). Structure, regulation and function of PKB/AKT – a major therapeutic target. Biochim Biophys Acta.

[B27] Corbould A, Zhao H, Mirzoeva S, Aird F, Dunaif A (2006). Enhanced mitogenic signaling in skeletal muscle of women with polycystic ovary syndrome. Diabetes.

[B28] Moller JT, Cluitmans P, Rasmussen LS, Houx P, Rasmussen H, Canet J, Rabbitt P, Jolles J, Larsen K, Hanning CD (1998). Long-term postoperative cognitive dysfunction in the elderly ISPOCD1 study. ISPOCD investigators. International Study of Post-Operative Cognitive Dysfunction. Lancet.

[B29] Ancelin ML, de Roquefeuil G, Ledesert B, Bonnel F, Cheminal JC, Ritchie K (2001). Exposure to anaesthetic agents, cognitive functioning and depressive symptomatology in the elderly. Br J Psychiatry.

[B30] Greenwood CE, Winocur G (2005). High-fat diets, insulin resistance and declining cognitive function. Neurobiol Aging.

[B31] Winocur G, Greenwood CE (2005). Studies of the effects of high fat diets on cognitive function in a rat model. Neurobiol Aging.

